# Impact of Insurance Coverage on Outcomes in Primary Breast Sarcoma

**DOI:** 10.1155/2018/4626174

**Published:** 2018-03-15

**Authors:** Julie L. Koenig, C. Jillian Tsai, Katherine Sborov, Kathleen C. Horst, Erqi L. Pollom

**Affiliations:** ^1^Department of Radiation Oncology, Stanford University Medical Center, 875 Blake Wilbur Drive, Stanford, CA 94305-5847, USA; ^2^Department of Radiation Oncology, Memorial Sloan Kettering Cancer Center, 1275 York Ave, New York, NY 10065, USA

## Abstract

Private insurance is associated with better outcomes in multiple common cancers. We hypothesized that insurance status would significantly impact outcomes in primary breast sarcoma (PBS) due to the additional challenges of diagnosing and coordinating specialized care for a rare cancer. Using the National Cancer Database, we identified adult females diagnosed with PBS between 2004 and 2013. The influence of insurance status on overall survival (OS) was evaluated using the Kaplan–Meier estimator with log-rank tests and Cox proportional hazard models. Among a cohort of 607 patients, 67 (11.0%) had Medicaid, 217 (35.7%) had Medicare, and 323 (53.2%) had private insurance. Compared to privately insured patients, Medicaid patients were more likely to present with larger tumors and have their first surgical procedure further after diagnosis. Treatment was similar between patients with comparable disease stage. In multivariate analysis, Medicaid (hazard ratio (HR), 2.47; 95% confidence interval (CI), 1.62–3.77; *p* < 0.001) and Medicare (HR, 1.68; 95% CI, 1.10–2.57; *p*=0.017) were independently associated with worse OS. Medicaid insurance coverage negatively impacted survival compared to private insurance more in breast sarcoma than in breast carcinoma (interaction *p* < 0.001). In conclusion, patients with Medicaid insurance present with later stage disease and have worse overall survival than privately insured patients with PBS. Worse outcomes for Medicaid patients are exacerbated in this rare cancer.

## 1. Introduction

Breast sarcomas are rare neoplasms that represent less than 5% of soft-tissue sarcomas and less than 1% of breast malignancies [1]. They develop de novo (primary) or are associated with prior radiation therapy or chronic lymphedema (secondary).

Management of rare cancers including breast sarcoma is challenging due to limited case numbers and lack of prospective clinical trials. Current recommendations for primary breast sarcoma are derived from small retrospective studies and extrapolated from the treatment of nonbreast soft-tissue sarcomas. Given the complexity of management and paucity of data, cases should be managed by multidisciplinary teams with expertise in sarcoma [2]. However, accessing these teams can be logistically challenging [3] and costly.

Health insurance coverage affects access to cancer care and has important clinical consequences. Insurance coverage influences whether patients undergo recommended cancer screening [4], receive appropriate and timely treatment, and participate in clinical trials [5]. Medicaid-insured patients tend to present at a more advanced stage [6, 7], with worse overall survival in breast carcinoma [8]. With more than 62 million people covered by Medicaid in the United States in 2016, it is vital to understand how cancer outcomes vary across health insurance types [9]. We hypothesized that nonprivate insurance status would be associated with worse outcomes in primary breast sarcoma due to the additional challenges of diagnosing and coordinating specialized care for a rare cancer.

## 2. Methods

### 2.1. Data Source

We performed a retrospective cohort study using the National Cancer Database (NCDB) 2014 Participant User File. This study was exempt from review by our institutional review board. The NCDB is a joint program of the Commission on Cancer (CoC) of the American College of Surgeons and the American Cancer Society (ACS) and is a hospital-based registry with data from more than 1,500 CoC-accredited hospitals. It includes information about demographics, disease stage, comorbidity, and the first course of treatment for 70% of newly diagnosed cancer cases in the United States. The CoC and American Cancer Society have not verified and are not responsible for the analytic or statistical methodology used or for the conclusions drawn from these data.

### 2.2. Patient Selection

We identified female patients 18 years and older who were diagnosed with histologically confirmed malignant breast sarcoma in 2004–2013 (International Classification of Diseases for Oncology-3 (ICD-O-3) site C50.0–50.9; ICD-O-3 histology 8800, 8801, 8802, 8810, 8830, 8831, 8850, 8851, 8852, 8854, 8890, 8894, 8930, 8940, 8990, 9120, 9130, 9180, 9220, and 9580) [10]. We excluded patients diagnosed in 2014 because they have incomplete follow-up. We included patients with breast sarcoma as their first or only cancer diagnosis to limit our analysis to patients with primary breast sarcoma. We excluded patients with unknown insurance status, no insurance (*n* = 41), and other government insurance (*n* = 8) due to low numbers (Table 1).

### 2.3. Covariates

Relevant patient, facility, and tumor characteristics were obtained from the database (Table 2). Insurance coverage was categorized as private/managed care, Medicaid, or Medicare based on the patient's primary insurance carrier at the time of diagnosis and/or treatment. The reporting facility cancer program type was dichotomized into academic programs including National Cancer Institute- (NCI-) designated comprehensive cancer centers and nonacademic programs. Distance from reporting facility was based on the shortest distance between the patient's residence and the reporting facility. Facility cancer program type and patient geographic location are not available from the NCDB for patients aged below 40 years to protect patient privacy. The stage was assigned according to the 7th edition of the American Joint Committee on Cancer Staging Manual [11]. Patients with no regional lymph nodes examined were considered N0 when defining the stage.

### 2.4. Treatment Characteristics

The primary surgical procedure was categorized into no surgery, breast-conserving surgery (BCS), mastectomy without reconstruction, mastectomy with reconstruction, and mastectomy not otherwise specified (NOS). Treatment was defined as no definitive treatment, surgery alone, surgery and radiation therapy (RT), and other. The “other” category included patients who received chemotherapy alone, RT alone, chemotherapy and RT, or unknown. Additional characteristics are listed in Table 3.

### 2.5. Statistical Analysis

Pearson chi-square tests were used to determine associations between insurance type and demographic, tumor, and treatment characteristics. Wilcoxon and Kruskal–Wallis tests were used to compare median age and time from diagnosis to first surgical procedure between insurance types. Differences in overall survival, defined as time to death or last contact after diagnosis, were assessed using the Kaplan–Meier estimator with log-rank testing. Univariate and multivariate Cox proportional hazard models were used to evaluate the impact of insurance type on survival. Covariates in the multivariate model were selected a priori and included insurance type, age, race, year of diagnosis, comorbidity score, income, residence environment type, distance from reporting facility, educational attainment, angiosarcoma histology, tumor size, lymph node status, metastatic status, grade, and treatment. All tests were two-sided with an alpha value of 0.05. Statistical analyses were performed using STATA/SE software (version 14.0, StataCorp, College Station, TX).

## 3. Results

### 3.1. Patient, Tumor, and Treatment Characteristics

We identified 607 patients who met our inclusion criteria (Table 1). 323 (53.2%) patients had private/managed care insurance, 67 (11.0%) had Medicaid, and 217 (35.7%) had Medicare. Baseline demographic and tumor characteristics are presented in Table 2.

Medicaid and privately insured patients were similar in age (median 50 and 52 years, resp.) and younger than Medicare patients (median 76 years). Medicaid-insured patients were more likely to be black and live in zip codes with lower median household income and educational attainment. Medicaid patients were less likely to have comorbid conditions and more likely to have large tumors, with 31.3% of Medicaid patients presenting with tumors greater than 10 cm in size.

Baseline treatment characteristics are presented in Table 3. The time from diagnosis to first surgical procedure was shorter for patients with private insurance than for those who were insured by Medicaid or Medicare. Privately insured patients were more likely to have breast-conserving surgery (BCS; 34.4%) and less likely to have a mastectomy (58.2%) compared to Medicare (22.6% and 64.5%, resp.) and Medicaid patients (23.9% and 74.6%, resp.). There was no difference in receipt of BCS between Medicaid (48.0%) and privately insured patients (44.0%) with stage I/II primary breast sarcoma (PBS; *P*=0.43). Among patients who had a mastectomy and known surgery details, privately insured patients were more likely to have reconstruction (20.5%) compared to Medicaid-insured (12.9%) and Medicare-insured (6.2%) patients.

Medicaid-insured patients were more likely to undergo surgery, radiation, and chemotherapy. Receipt of RT was not significantly different between Medicaid and privately insured patients presenting with high-grade tumors (42.9% versus 51.3%, resp.; *p*=0.58) or large tumors (>5 cm; 50.0% versus 51.2%, resp.; *p*=0.07) who would be most likely to benefit from adjuvant RT [12]. Among patients undergoing lumpectomy, receipt of RT was not significantly different between Medicaid-insured (50.0%), privately insured (41.4%), and Medicare-insured (34.7%) patients (*p*=0.38).

### 3.2. Survival Analysis

**Figure 1:**
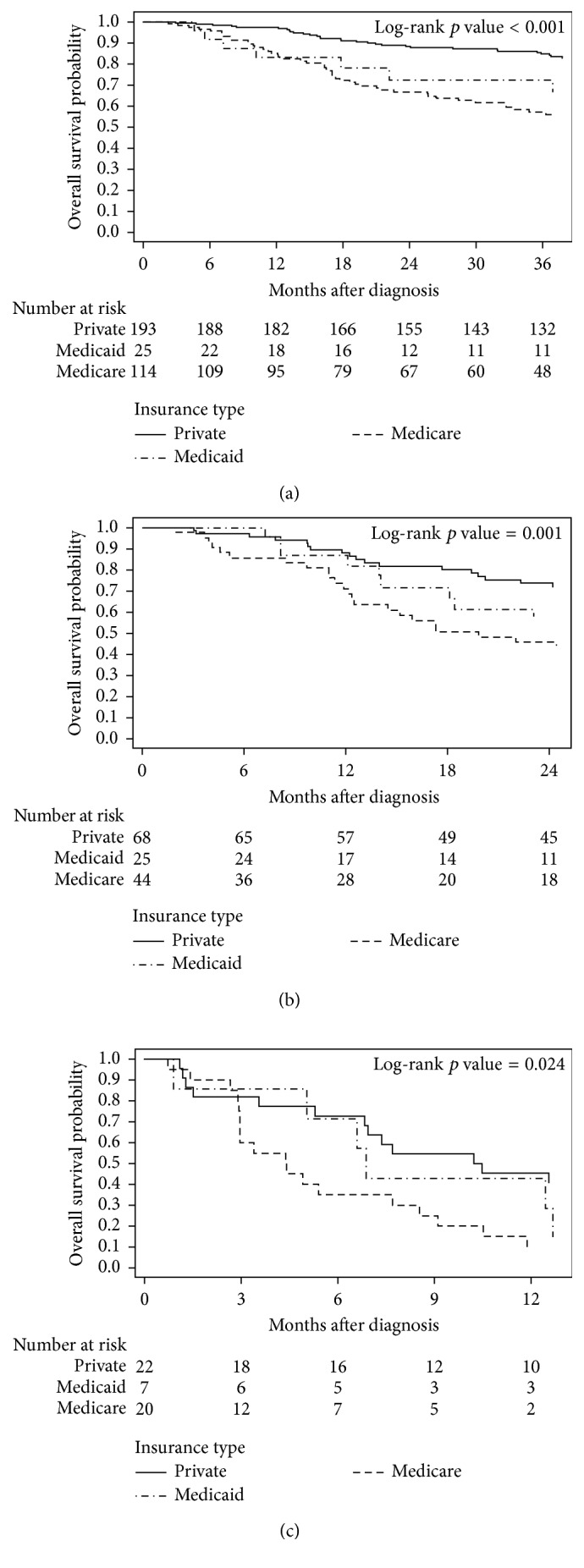
Kaplan–Meier plots of overall survival stratified by insurance coverage type (private insurance, Medicaid, and Medicare) for patients with (a) stage I/II, (b) stage III, and (c) stage IV primary breast sarcomas.

Median follow-up time from the date of diagnosis to the date of death or last contact was 42 months (interquartile range (IQR), 21 to 77 months), 27 months (IQR, 12 to 48 months), and 7 months (IQR, 3 to 13 months) for stage I/II (Figure 1), III (Figure 1), and IV (Figure 1) PBS, respectively. For patients with nonmetastatic PBS, those who were privately insured had greater estimated 3-year OS compared to those with Medicaid and Medicare (79.4%, 54.9%, and 51.1%, resp.; Figures 1 and 1).

On univariate analysis, Medicaid and Medicare insurance were associated with increased hazard of death compared to private insurance (Table 4). After controlling for sociodemographic, tumor, and treatment characteristics in multivariate analysis, Medicaid and Medicare insurance remained independently associated with worse OS (Table 4).

### 3.3. Sensitivity Analysis

Our findings did not change when we created another model additionally adjusting for facility type, thereby excluding patients aged below 40 years; Medicaid and Medicare patients again had worse survival outcomes compared to privately insured patients. Treatment at nonacademic facility type was associated with worse OS relative to treatment at an academic facility (HR, 1.52; 95% CI, 1.12–2.07; *p*=0.008) in this model.

To evaluate whether the impact of insurance on outcomes was greater among patients with a rare cancer like breast sarcoma compared to breast carcinoma, we constructed another model that included patients with breast carcinoma to look at the interaction between cancer type (breast sarcoma versus breast carcinoma) and insurance category (Medicaid versus private) (Table 5). Compared to sarcoma patients, breast carcinoma patients with Medicaid had less of a survival detriment relative to privately insured patients (interaction *p* < 0.001). Cancer type did not significantly modify the effect of Medicare versus private insurance on overall survival.

## 4. Discussion

Using a nationwide database of women with primary breast sarcoma (PBS), we found that patients who were privately insured had better survival than those who had nonprivate insurance. We also found that the Medicaid-related health disparity was greater in the setting of a rare cancer.

Our findings contribute to the growing literature highlighting the importance of insurance status in cancer outcomes and quality of care. While previous studies have explored the relationship between insurance status and outcomes in other common cancers including breast cancer [6–8], our study addresses this question in patients with breast sarcoma, a rare cancer. We hypothesized that insurance coverage would greatly impact the presentation, management, and outcomes of a rare tumor due to the difficulty of diagnosis and importance of early intervention and specialized care. We found a notable difference in outcome depending on private versus nonprivate insurance coverage among patients with PBS. Those patients who had nonprivate insurance coverage in our study had an almost twofold higher risk of mortality compared to patients who had private insurance coverage. We also found that Medicaid insurance coverage, but not Medicare, differentially impacted outcomes in breast sarcoma compared to breast carcinoma. This suggests that factors associated with worse outcomes for Medicaid-insured patients are exacerbated in a rare cancer.

There are many factors that could lead to worse outcomes in Medicaid patients ranging from the consequences of Medicaid insurance policies to the socioeconomic factors driving patients to be insured by Medicaid. While insurance coverage reduces disparities in cancer outcomes, it does not completely eliminate the effects of social determinants of health such as race and socioeconomic status [13]. Whites benefit more from private insurance than do blacks and Hispanics [14]. Racial and ethnic minorities make up a disproportionate amount of Medicaid beneficiaries [15] and have independently worse cancer outcomes [16]. Health insurance coverage also depends on other upstream socioeconomic factors known to influence cancer outcomes: social support, income, education, and health literacy including knowledge about screening. Adults who are privately insured tend to be of higher average health literacy than adults who are uninsured or insured by Medicare or Medicaid [17]. Individuals with low health literacy are more likely to delay seeking care and have more difficulty finding providers [18]. In our study, privately insured patients underwent more breast reconstruction which may be due to better access to plastic surgeons and even better treatment-related decision-making, as shown in the breast carcinoma setting [19]. Medicaid patients tended to be non-white and to reside in areas of lower income and educational attainment, so these factors likely contributed to the worse outcomes of Medicaid patients that we observed. Worse outcomes in the Medicare population are most likely driven by older age and the presence of significant comorbidities that qualify younger patients (<65 years) for Medicare.

We found that Medicaid-insured patients with breast sarcoma were more likely to present with large, advanced stage tumors. This association builds upon previous studies that have found that patients with nonprivate insurance have an increased likelihood of presenting with advanced breast, prostate, lung, colorectal, head and neck, liver, and pancreatic cancers compared to those with private insurance [7, 8, 20, 21]. Medicaid-insured patients may present with more advanced disease due to lower rates of cancer screening [22], less frequent interactions with primary care [6], and cancer serving as a Medicaid-qualifying event. Patients enrolled in Medicaid after their cancer diagnosis present with more advanced disease [23] and have worse outcomes [24, 25] compared to those covered by Medicaid prior to diagnosis. Nonprivately insured patients are also vulnerable to a variety of patient and health system-related delays in presentation, diagnosis, and referral to specialty centers [22]. In our study, the time between diagnosis and first surgical procedure was longer for Medicaid-insured patients, which may reflect the difficulty of coordinating care for patients with limited insurance coverage and more advanced disease. Delays between diagnosis and treatment of breast cancer have been shown to be associated with inferior cancer outcomes [26].

Commensurate with more advanced stage tumors, Medicaid patients underwent more aggressive locoregional management of PBS. However, privately insured and Medicaid-insured patients with comparable disease stage received similar treatment. Many Medicaid patients were likely ineligible for breast-conserving surgery (BCS) due to presentation with very large (>10 cm) tumors. We found similar rates of BCS among privately insured and Medicaid-insured patients with stage I/II disease. Receipt of radiation therapy (RT) was also similar between privately insured and Medicaid-insured patients undergoing lumpectomy or presenting with large or high-grade tumors. Given the similarity in treatment, presentation with advanced disease may be a more important driver of poor outcomes in Medicaid patients.

After adjusting for sociodemographic, tumor, and treatment characteristics, Medicaid and Medicare patients continued to have worse outcomes than privately insured patients. Furthermore, the health disparity associated with Medicaid insurance was significantly worse in patients with breast sarcoma versus breast carcinoma. Medicaid insurance may be a greater challenge for patients with rare cancers because of limited access to centers with specialists [27] and multidisciplinary teams that can coordinate care and expedite treatment [28]. Specialized centers have been shown to have better outcomes for rare cancers [29], and some recommend large soft-tissue sarcomas be treated exclusively at high-volume centers [30].

Access to specialists and insurance network adequacy is a concern among patients who have Medicaid insurance. Medicaid-managed care plans can limit access to providers and “off-label” drugs, including chemotherapies [31]. Medicaid patients, who are more likely to be underrepresented in clinical trials based on race and ethnicity [32], face additional obstacles for receiving novel treatments because Medicaid does not always cover routine medical costs associated with clinical trials [15]. Medicaid patients also have limited access to high quality care because specialty providers are less likely to accept patients with Medicaid [15]. This is in part due to Medicaid reimbursement rates being well below the rates paid by Medicare and private insurance [33]. Even safety net hospitals may try to attract privately insured patients to increase payer mix and revenue [34]. Medicaid patients are additionally susceptible to coverage gaps and limited healthcare access due to insurance “churning,” the involuntary movement between insurance plans [35]. It is estimated that more than 25 million Americans per year will move from Medicaid to subsidized exchange plans or will become ineligible for subsidized programs altogether [35]. These frequent lapses and transitions in coverage likely contribute to Medicaid patients presenting with more advanced disease and diminish the health benefits of insurance coverage [22].

Medicare insurance did not differentially impact outcomes in breast sarcoma compared to breast carcinoma. This may suggest that the survival detriment associated with Medicare is related to the agedness and higher comorbidity of Medicare patients more than the healthcare access issues that might be worse with a rare cancer. Medicare patients have better access to care and higher provider reimbursements than Medicaid patients do. Consistent with this, Medicare patients have been shown to benefit from improved cancer treatment and survival over time, while survival disparities are worsening for Medicaid patients [36].

In this study, treatment at academic and NCI-designated cancer centers was independently associated with better overall survival in a subset of patients with available facility type. While we did not find that privately insured patients were more likely to be treated at academic centers, our analysis is limited by the large percentage of patients with unknown treatment facility type, which the NCDB suppresses for patients aged below 40 years for privacy reasons. Additionally, we were unable to distinguish NCI-designated comprehensive cancer centers from academic centers, which represent the majority of safety net hospitals caring for the underinsured [37]. Nevertheless, our data may suggest that better outcomes in privately insured patients are driven by other factors in addition to treatment facility type and access to multidisciplinary teams and specialty care.

Our finding of worse outcomes in Medicaid-insured patients does not necessarily suggest that Medicaid itself is inferior to private insurance because Medicaid is designed to cover poorer patients who tend to have more advanced disease. Almost half of the Medicaid beneficiaries with cancer enroll in Medicaid after they are diagnosed with cancer when they already have advanced disease [24, 25]. Intensive treatment may not be able to compensate for late presentation. While we attempted to control for sociodemographic, tumor, and treatment factors, it is beyond the scope of the NCDB to completely differentiate the health consequences of Medicaid policies and the socioeconomic factors driving patients to be insured by Medicaid in the first place. Rather this study highlights that the insurance policy and/or socioeconomic factors leading to worse outcomes in patients insured by Medicaid are exacerbated in a rare cancer such as breast sarcoma. Improving access to high quality care and addressing the barriers that Medicaid patients face are potential ways to alleviate the impact that socioeconomic factors have on health outcomes.

Our study has multiple limitations including its retrospective design and reliance on the content and accuracy of data included in the NCDB. While we attempted to control for observed confounders, we could not control for unmeasured confounders such as baseline performance status, patient preference, and molecular data. Additionally, we were unable to account for the clinical heterogeneity of sarcomas, which exists even among tumors of the same histologic subtype [38]. Furthermore, our analysis is limited by the insurance coverage data available in the NCDB, which is the primary payer at the time of diagnosis or treatment. The NCDB captures a single primary payer, but some patients may have dual insurance coverage or may transition between insurance plans during treatment. While the NCDB provides the opportunity to assess outcomes of patients receiving care in community settings where multidisciplinary teams and sarcoma experts may be less accessible, we recognize that CoC-accredited facilities are enriched for large, urban hospitals with strong oncology services [39]. Despite these limitations, the NCDB is a good resource for studying rare cancers such as PBS because prospective studies do not exist and the NCDB provides information on a larger scale than existing retrospective, single-institution studies.

In conclusion, we show that privately insured patients with PBS present with earlier stage disease and have better outcomes. Further work is warranted to address the cultural and socioeconomic factors that lead to such health disparities.

## Figures and Tables

**Table 1:** Cohort selection.

		No.	%
1	Total breast cancer cases diagnosed from 2004 to 2014	2,246,280	100.00%
2	Limit to female patients 18 years and older with histologically confirmed primary invasive breast sarcoma diagnosed from 2004 to 2013	741	0.03%
3	Exclude patients whose diagnosis date precedes reference date to ensure data completeness	723	0.03%
4	Exclude patients who were diagnosed at reporting facility but did not receive any treatment at that facility	696	0.03%
5	Exclude patients with unknown vital status or unknown follow-up; exclude patients diagnosed at autopsy	683	0.03%
6	Exclude if insurance status is unknown	656	0.03%
7	Limit to patients with private, Medicaid, or Medicare insurance	607	0.03%

**Table 2:** Baseline patient and tumor characteristics by insurance coverage type.

Patient and tumor characteristics	Private	Medicaid	Medicare	
	No. (%)	No. (%)	No. (%)	*P* ^*a*^
Total^b^	323 (53.2%)	67 (11.0%)	217 (35.7%)	—
Age (years)				<0.001
18–44	95 (29.4%)	20 (29.9%)	<11	—
45–54	101 (31.3%)	29 (43.3%)	<11	—
55–64	97 (30.0%)	≥11	13 (6.0%)	—
≥65	30 (9.3%)	<11	192 (88.5%)	—
Race				0.001
White	249 (77.1%)	40 (59.7%)	182 (83.9%)	—
Black	50 (15.5%)	≥11	≥11	—
Other/unknown	24 (7.4%)	<11	<11	—
Year of diagnosis				0.506
2004-2005	76 (23.5%)	≥11	40 (18.4%)	—
2006-2007	64 (19.8%)	<11	40 (18.4%)	—
2008-2009	61 (18.9%)	15 (22.4%)	54 (24.9%)	—
2010-2011	66 (20.4%)	15 (22.4%)	42 (19.4%)	—
2012-2013	56 (17.3%)	14 (20.9%)	41 (18.9%)	—
Charlson–Deyo score				<0.001
0	286 (88.5%)	≥11	149 (68.7%)	—
≥1	37 (11.5%)	<11	68 (31.3%)	—
Residence type				0.419
Metropolitan	280 (86.7%)	54 (80.6%)	183 (84.3%)	—
Urban/rural	34 (10.5%)	11 (16.4%)	31 (14.3%)	—
Income^c^				0.026
<$38,000	51 (15.8%)	20 (29.9%)	41 (18.9%)	—
≥$38,000	269 (83.3%)	45 (67.2%)	175 (80.6%)	—
Educational attainment (% without HSD)^c^				<0.001
≥13%	130 (40.2%)	43 (64.2%)	88 (40.6%)	—
<13%	190 (58.8%)	22 (32.8%)	129 (59.4%)	—
Facility location^d^				<0.001
Northeast	58 (18.0%)	≥11	43 (19.8%)	—
South	87 (26.9%)	15 (22.4%)	76 (35.0%)	—
Central	58 (18.0%)	15 (22.4%)	61 (28.1%)	—
West	51 (15.8%)	<11	≥11	—
Unknown	69 (21.4%)	19 (28.4%)	<11	—
Facility cancer program type^d^				<0.001
Academic/research^e^	101 (31.3%)	21 (31.3%)	≥11	—
Nonacademic	153 (47.4%)	27 (40.3%)	152 (70.0%)	—
Unknown	69 (21.4%)	19 (28.4%)	<11	—
Distance from facility				0.087
≤50 miles	289 (89.5%)	≥11	198 (91.2%)	—
>50 miles	31 (9.6%)	<11	19 (8.8%)	—
Angiosarcoma				0.858
Yes	111 (34.4%)	21 (31.3%)	71 (32.7%)	—
No	212 (65.6%)	46 (68.7%)	146 (67.3%)	—
Stage^f^				0.010
I/II	193 (59.8%)	25 (37.3%)	114 (52.5%)	—
III	68 (21.1%)	25 (37.3%)	44 (20.3%)	—
IV	22 (6.8%)	<11	20 (9.2%)	—
Unknown	40 (12.4%)	<11	39 (18.0%)	—
Tumor size category				<0.001
T1: ≤5 cm	166 (51.4%)	≥11	102 (47.0%)	—
T2: >5 cm	127 (39.3%)	48 (71.6%)	90 (41.5%)	—
Unknown	30 (9.3%)	<11	25 (11.5%)	—
Node status^g^				0.435
N0	135 (41.8%)	33 (49.3%)	85 (39.2%)	—
N+	11 (3.4%)	<11	<11	—
No nodes examined/unknown	177 (54.8%)	≥11	≥11	—
Metastatic status^h^				0.659
M0	284 (87.9%)	55 (82.1%)	183 (84.3%)	—
M1	24 (7.4%)	<11	20 (9.2%)	—
Unknown	15 (4.6%)	<11	14 (6.5%)	—
Grade				0.096
G1: well differentiated	58 (18.0%)	<11	29 (13.4%)	—
G2: moderately differentiated	44 (13.6%)	<11	22 (10.1%)	—
G3: poorly/undifferentiated/anaplastic	150 (46.4%)	42 (62.7%)	108 (49.8%)	—
Unknown	71 (22.0%)	11 (16.4%)	58 (26.7%)	—

In order to protect patient identity, some categories are combined, unknown categories with few patients are not shown, and cells with fewer than 11 patients are hidden; ^a^Pearson's chi-squared *p* value; ^b^percentage of total cohort (*n* = 607); ^c^derived from patient zip code and 2012 American Community Survey data from years 2008–2012; ^d^not available for patients <40 years old; ^e^includes NCI-designated comprehensive cancer centers; ^f^patients with no nodes examined were considered N0 when defining the stage; ^g^presence or absence of any involved regional lymph nodes at diagnosis; ^h^presence or absence of any distant metastases at diagnosis; abbreviations: HSD, high school degree.

**Table 3:** Baseline treatment characteristics by insurance coverage type.

Treatment Characteristics	Private	Medicaid	Medicare	
	No. (%)	No. (%)	No. (%)	*P* ^a^
Total	323 (53.2%)	67 (11.0%)	217 (35.7%)	—
Primary surgical procedure				<0.001
No surgery	22 (6.8%)	<11	26 (12.0%)	—
BCS	111 (34.4%)	16 (23.9%)	49 (22.6%)	—
Mastectomy without reconstruction	124 (38.4%)	27 (40.3%)	91 (41.9%)	—
Mastectomy with reconstruction	32 (9.9%)	<11	<11	—
Mastectomy NOS	32 (9.9%)	19 (28.4%)	≥11	—
Surgical margins				0.217
Negative	272 (84.2%)	58 (86.6%)	171 (78.8%)	—
Positive^b^	18 (5.6%)	<11	13 (6.0%)	—
NA/unknown	33 (10.2%)	<11	33 (15.2%)	—
Receipt of RT				0.004
No	188 (58.2%)	33 (49.3%)	150 (69.1%)	—
Yes	131 (40.6%)	31 (46.3%)	66 (30.4%)	—
Receipt of chemotherapy				0.021
No	221 (68.4%)	43 (64.2%)	173 (79.7%)	—
Yes	94 (29.1%)	23 (34.3%)	39 (18.0%)	—
Treatment				<0.001
No definitive tx	12 (3.7%)	<11	17 (7.8%)	—
Surgery alone	124 (38.4%)	22 (32.8%)	112 (51.6%)	—
Surgery and RT	126 (39.0%)	31 (46.3%)	60 (27.6%)	—
Other^c^	61 (18.9%)	≥11	28 (12.9%)	—
	Median (IQR)	Median (IQR)	Median (IQR)	*P* ^*d*^
Days from diagnosis to surgery	15.0 (0.0, 33.0)	27.5 (8.0, 44.0)	19.0 (0.0, 35.0)	0.017

In order to protect patient identity, some categories are combined, unknown categories with few patients are not shown, and cells with fewer than 11 patients are hidden; ^a^Pearson's chi-squared *p* value; ^b^includes residual tumor that is microscopic, macroscopic, and not otherwise specified; ^c^includes patients who received chemotherapy alone (*n* = 76), RT alone (*n* < 11), chemotherapy and RT (*n* < 11), and unknown (*n* = 16); ^d^Kruskal–Wallis *p* value; abbreviations: BCS, breast-conserving surgery; NOS, not otherwise specified; NA, not applicable; RT, radiation therapy; tx, treatment; IQR, interquartile range.

**Table 4:** Univariate and multivariate Cox proportional hazard models.

Variable	Univariate analysis		Multivariate analysis	
	HR (95% CI)	*p*	HR (95% CI)	*p*
Insurance coverage				
Private	Reference		Reference	
Medicaid	3.01 (2.04–4.45)	<0.001	2.47 (1.62–3.77)	<0.001
Medicare	2.55 (1.94–3.33)	<0.001	1.68 (1.10–2.57)	0.017
Age (years)				
18–44	Reference		Reference	
45–54	1.34 (0.87–2.06)	0.187	1.20 (0.76–1.89)	0.435
55–64	1.34 (0.86–2.08)	0.194	1.52 (0.95–2.43)	0.081
≥65	2.40 (1.65–3.50)	<0.001	2.03 (1.20–3.43)	0.008
Race				
White	Reference		Reference	
Black	0.90 (0.63–1.27)	0.543	0.65 (0.43–0.98)	0.040
Other/unknown	0.43 (0.23–0.81)	0.009	0.45 (0.24–0.87)	0.018
Year of diagnosis				
2004-2005	Reference		Reference	
2006-2007	0.80 (0.54–1.18)	0.256	0.70 (0.47–1.06)	0.095
2008-2009	1.31 (0.92–1.87)	0.130	1.04 (0.71–1.51)	0.843
2010-2011	1.27 (0.87–1.86)	0.220	0.68 (0.44–1.03)	0.066
2012-2013	0.94 (0.60–1.49)	0.802	0.74 (0.46–1.18)	0.207
Charlson–Deyo score				
0	Reference		Reference	
1	1.19 (0.86–1.66)	0.291	1.13 (0.77–1.63)	0.535
≥2	3.28 (1.67–6.42)	0.001	1.40 (0.68–2.92)	0.363
Income				
<$38,000	Reference		Reference	
≥$38,000	0.77 (0.57–1.05)	0.099	0.95 (0.65–1.39)	0.795
Residence type				
Metropolitan	Reference		Reference	
Urban/rural	1.21 (0.85–1.72)	0.282	1.01 (0.67–1.53)	0.956
Distance from facility				
≤50 miles	Reference		Reference	
>50 miles	0.93 (0.61–1.43)	0.741	0.72 (0.45–1.16)	0.179
Educational attainment (% without HSD)				
≥13%	Reference		Reference	
<13%	0.87 (0.67–1.11)	0.255	0.89 (0.66–1.19)	0.415
Angiosarcoma				
Yes	Reference		Reference	
No	0.96 (0.74–1.24)	0.755	0.82 (0.62–1.10)	0.183
Tumor size category				
T1: ≤5 cm	Reference		Reference	
T2: >5 cm	3.26 (2.47–4.30)	<0.001	3.66 (2.66–5.02)	<0.001
Unknown	1.72 (1.08–2.74)	0.022	1.56 (0.95–2.54)	0.079
Node status				
N0	Reference		Reference	
N+	1.54 (0.75–3.16)	0.239	1.43 (0.67–3.06)	0.348
No nodes examined/unknown	0.98 (0.76–1.26)	0.880	0.92 (0.70–1.23)	0.586
Metastatic status				
M0	Reference		Reference	
M1	7.20 (5.19–10.00)	<0.001	7.19 (4.69–11.03)	<0.001
Grade				
G1: well differentiated	Reference		Reference	
G2: moderately differentiated	1.36 (0.78–2.38)	0.280	1.49 (0.83–2.69)	0.181
G3: poorly, undifferentiated, or anaplastic	2.67 (1.74–4.12)	<0.001	2.23 (1.39–3.58)	0.001
Unknown	2.04 (1.25–3.30)	0.004	1.89 (1.14–3.13)	0.014
Treatment summary				
No definitive treatment	Reference		Reference	
Surgery alone	0.31 (0.18–0.53)	<0.001	0.20 (0.11–0.37)	<0.001
Surgery and RT	0.27 (0.15–0.47)	<0.001	0.12 (0.06–0.23)	<0.001
Other/unknown	0.60 (0.34–1.07)	0.083	0.22 (0.11–0.43)	<0.001

Abbreviations: HR, hazard ratio; CI, confidence interval; HSD, high school degree; RT, radiation therapy.

**Table 5:** Multivariate Cox proportional hazard models of the effect of insurance status on overall survival in patients with breast sarcoma versus carcinoma.

	Multivariate analysis^a^	
	HR (95% CI)	*p*
Interaction between insurance status and breast cancer type^b^		
Medicaid versus private; sarcoma versus carcinoma	—	<0.001
Medicare versus private; sarcoma versus carcinoma	—	0.912
Breast cancer type		
Carcinoma: Medicaid versus private (reference)	1.69 (1.66–1.72)	<0.001
Sarcoma: Medicaid versus private (reference)	2.48 (1.63–3.78)	<0.001

^a^Adjusted for age, race, year of diagnosis, comorbidity, income, location, distance from treatment facility, educational attainment, tumor size, node status, metastatic status, tumor grade, and treatment; ^b^*p* value for the interaction tests whether there is a significant difference in the HRs for patients with Medicaid/Medicare insurance relative to private insurance in breast sarcoma versus breast carcinoma; abbreviations: HR, hazard ratio; CI, confidence interval.
